# Ecology and Fungicolous Lifestyle of *Hypomyces aurantius* Associated with the Newly Recorded Host *Lyophyllum littoralis*

**DOI:** 10.3390/jof12090632

**Published:** 2026-08-24

**Authors:** Halide Karabıyık, İsmail Acar

**Affiliations:** 1 Department of Food Processing, Arda Vocational School, Trakya University, Edirne 22100, Türkiye; 2 Department of Organic Agriculture, Başkale Vocational School, Van Yüzüncü Yıl University, Van 65080, Türkiye; ismailacar@yyu.edu.tr

**Keywords:** fungicolous, mycoparasitism, new records, phylogeny, Türkiye

## Abstract

Despite developing on other fungi and forming complex ecological interactions, fungicolous fungi are among the understudied groups. This study examined the fungicolous fungi *Hypomyces aurantius* and its host species, *Lyophyllum littoralis*, in detail using morphological and molecular data. Both species are new records for the Turkish mycobiota. The macroscopic and microscopic characteristics of both species were evaluated in depth, and morphological descriptions were corroborated by molecular analyses based on the nuclear ITS region. It was observed that *H. aurantius* heavily colonised *L. littoralis*, a previously unreported host, causing significant softening and degradation of the host tissue. We investigated extracellular enzyme activities to determine the mycoparasitic activity of *H. aurantius* on the host fungus and its environmental survival strategies. We detected positive enzyme activities. These results suggest that *H. aurantius* is a potential mycoparasite. This study contributes to our understanding of fungal biodiversity in Türkiye and provides a foundation for understanding the ecology of supra-fungal fungi and fungicolous/mycoparasitic interactions. conclusions.

## 1. Introduction

Fungicolous fungi are specialised organisms that spend all or part of their life cycle on the mycelium, sporocarp or reproductive structures of other fungi. They can develop saprotrophic, commensal, necrotrophic or mycoparasitic life strategies, playing important roles in the structure of fungal communities, interspecies competition, sporocarp decomposition and nutrient cycling in natural ecosystems [1,2,3,4]. Macrofungal sporocarps, in particular, provide suitable, albeit short-lived, microhabitats for fungicolous fungi due to their high water and organic matter content. In recent years, the widespread use of molecular methods has revealed that macrofungal sporocarps harbour richer and more diverse host-associated fungal communities than previously thought. However, the taxonomic composition, host preferences, and ecological functions of these communities remain poorly understood [2,5,6].

Mycoparasitism, one of the life forms of fungi, is considered one of the most specialized examples of fungus–fungus interactions. Mycoparasitic fungi can recognize a suitable host through chemical signals, direct themselves toward the host hyphae to establish contact, and colonize host tissues with the help of various biochemical mechanisms. This process involves not only physical contact but also the combined action of hydrolytic enzymes capable of breaking down the cell wall, oxidative enzyme systems, and various secondary metabolites [7,8]. The degradation of the fungal cell wall’s major components and structural proteins is considered one of the key steps in successful colonization. Therefore, extracellular enzyme production serves as an important indirect indicator in the evaluation of the physiological characteristics of fungicolous fungi and their potential mycoparasitic life strategies [7].

*Hypomyces* (Fr.) Tul. & C. Tul. is one of the most important fungicolous genera in the Hypocreaceae family and includes species that develop on numerous macrofungal hosts worldwide. Demonstrating fungicolous and mycoparasitic characteristics across various macrofungus groups, particularly Boletales, Helotiales, Agaricales, Pezizales and Polyporales, *Hypomyces* (Fr.) Tul. & C. Tul. is an ascomycete genus comprising over 150 recognised species that spread as powdery textures and puffy crusts on fungal surfaces [9,10,11]. Members of the genus can cause discolouration, the formation of a superficial mycelial covering, deformation and, in later stages, tissue degradation in host sporocarps. These characteristics mean they are considered one of the most important biotic factors affecting the dynamics of natural fungal populations [1,12]. Taxa belonging to the genus have been reported to be associated with environments other than fungi, such as various plant substrates (e.g., leaves, wood, and bark), mosses, lichens, rocks and humus [13].

*Hypomyces* species exhibit remarkable diversity in their host preferences. While some species show a high degree of dependence on specific genera or families, others can grow on macrofungi belonging to different taxonomic groups. Therefore, new host records provide important information not only from a mycobiotic perspective but also in understanding the host adaptation, ecological resilience, and evolutionary relationships of the species [1]. Recent phylogenetic studies have revealed that the host spectrum and infection strategies of *Hypomyces* (anamorphic stage *Cladobotryum*) are more complex than previously thought [12,14]. Therefore, documenting new fungicolous relationships with morphological and molecular data contributes not only to updating species distributions but also to a better understanding of the ecology and evolution of fungicolous fungi [2]. Among these species, *Hypomyces aurantius* (anamorph: *Cladobotryum varium*) has been reported from a wide range of macrofungal hosts, including species of *Panellus*, *Flammulina*, *Morchella*, poroid fungi and several other agaricoid basidiomycetes. Although the species is regarded as having a relatively broad host range, host associations remain incompletely documented because many historical records lack molecular confirmation. Consequently, every well-documented host association contributes to a more accurate understanding of its ecological amplitude and host specificity [1,15,16,17].

*H. aurantius*, which has generally been recorded on fungi such as *Panellus*, the winter mushroom (*Flammulina velutipes* (Curtis) Singer), morels (*Morchella*) and poroid species [11,17,18], has not been reported on *Lyophyllum littoralis* to date. This study aimed to confirm, using morphological and molecular methods, the previously unreported fungal association between *H. aurantius* and *L. littoralis*. In this context, the macro- and micromorphological characteristics of both taxa were examined in detail; their molecular identities were confirmed using ITS sequences, and their phylogenetic positions were assessed. Furthermore, the cultural characteristics of *H. aurantius* and its extracellular enzyme production profile were determined, and the species’ fungicolous lifestyle and potential mycoparasitic strategies were evaluated from a physiological perspective. Thus, the study not only presents the first records of *H. aurantius* and *L. littoralis* for the Turkish mycobiota, but also contributes to the existing body of knowledge on the host relationships and ecological characteristics of *Hypomyces* species by revealing a previously unreported host relationship between these two species in the light of morphological, molecular and physiological data.

## 2. Materials and Methods

### 2.1. Sample Collection

**Figure 1:**
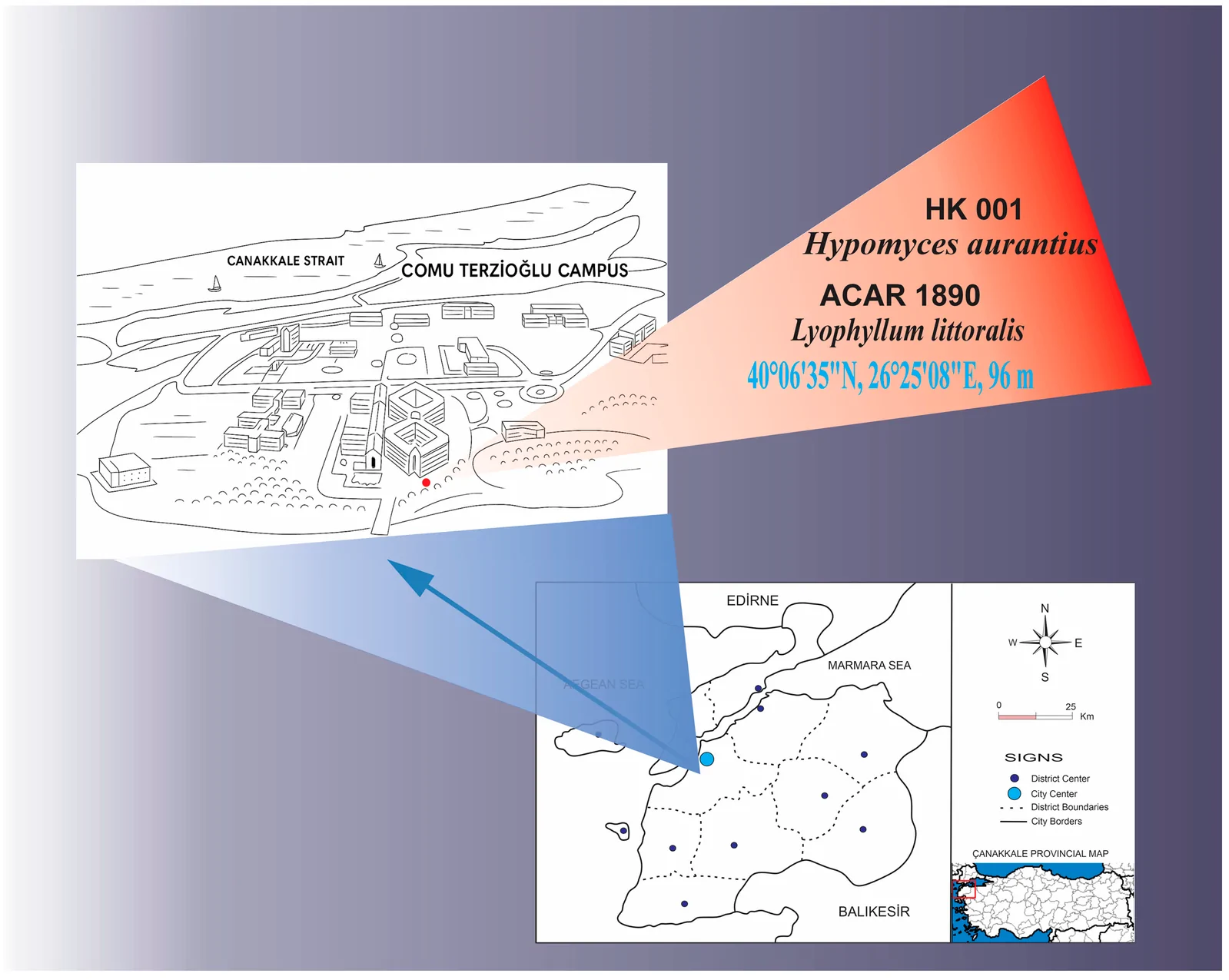
Map of the research area (Çanakkale, Türkiye).

The materials for this study, healthy macrofungus specimens bearing fungicolous microfungi, were collected in January 2025 from the vicinity of the Faculty of Science, Terzioğlu Campus, Çanakkale Onsekiz Mart University (Figure 1). The collected specimens were photographed in their natural environment, and environmental conditions and macromorphological characteristics (pileus, lamella, stipe, etc.) were examined in detail, with all details recorded. Specimens showing fungicolous growth were carefully placed in sterile bags, while healthy specimens were placed in separate boxes and quickly transported to the laboratory.

### 2.2. Isolation of Fungicolous Microfungi

The isolation of the fungicolous microfungi was performed by examining the macrofungi, which were brought to the laboratory in sterile bags, under a stereomicroscope. To this end, structures such as the microfungus’s mycelium and spore masses observed on the host fungus were transferred to PDA and MEA plates using sterile swabs. The inoculated plates were then incubated at 25 °C for seven days under a daylight cycle. After incubation, the growing fungicolous microfungi was obtained as a pure culture by isolating single spores. Stock cultures were obtained from this pure culture by transferring it to tubes containing slanted PDA agar medium, which were then stored in the microbiology laboratory at Trakya University Arda Vocational School.

### 2.3. Fungal Identification

Traditional taxonomic methods, combined with advanced molecular techniques, were employed to identify and classify host fungi and their associated fungicolous fungi. In this study, the macroscopic and microscopic characteristics of the samples were examined in detail, and the data obtained were evaluated by comparing them with ITS rDNA (nuclear internal transcribed spacer) gene sequences and phylogenetic analyses.

#### 2.3.1. Morphological Identification

Mycelium discs (5 mm) were extracted from the active growth tips of the Fungikol mushroom culture and inoculated onto plates of Potato Dextrose Agar (PDA, Merck, Darmstadt, Germany) and Malt Extract Agar (1.5% malt extract and 1.5% agar, Merck, Darmstadt, Germany). After incubation, the colonies were examined for their macroscopic and microscopic characteristics, and measurements were taken. Healthy macrofungus samples brought to the laboratory were also subjected to detailed micromorphological examination.

Specimens of both the host and the fungicolous fungi were examined under a stereomicroscope (Motic SMZ-171 Stereo Zoom Microscope, Motic Hong Kong Limited, Hong Kong, China) and a light microscope (Leica DM500, Leica Microsystems GmbH, Wetzlar, Germany). To reliably evaluate the microscopic characteristics of each structure (e.g., basidiospore, basidium, conidiospore and conidiogenic cell), measurements were taken at least 30 times. In addition, electron microscopy (SEM) images were used to identify the macrofungus specimen. To accurately and precisely present the obtained micromorphological findings, scientific drawings were prepared using the 64-bit CorelDRAW Graphics Suite 2022 (64-bit; Corel Corporation, Ottawa, ON, Canada), along with microphotographs.

The macro- and micro-morphological characteristics of the Fungicolous species were assessed based on the descriptions provided by Tulasne & Tulasne (1860) [19], Rogerson & Samuels (1993) [20], and Arnold & Yurchenko (2007) [21]. The host species, meanwhile, were assessed by comparison with the descriptions provided by Ballero & Contu (1990) [22], Contu (1998) [23], *Archivio Micologico* (2016) [24], and iNaturalist (2026) [25].

Following identification, *H. aurantius* was prepared as a fungarium specimen and preserved as a stock culture (code HK001) in the Microbiology Laboratory of Arda Vocational School, Trakya University. The dried basidiomata of *L. littoralis* were deposited in the fungarium of the Department of Biology, Faculty of Science, Van Yüzüncü Yıl University, under the personal fungarium number Acar1890. The collection, isolation, and micromorphological examination of the fungi were performed according to the procedures described by [26].

#### 2.3.2. Molecular Identification

Genomic DNA was extracted from the macrofungal samples used in this study following the CTAB protocol [27]. The nuclear ribosomal internal transcribed spacer (nrITS) region was amplified by polymerase chain reaction (PCR) from DNA extracted from samples HK001 and ACAR 1890. DNA isolation and PCR procedures were carried out in accordance with the protocols described in previous studies [28,29,30,31], thereby ensuring methodological consistency and reproducibility. For DNA isolation, the EurX GeneMATRIX Plant & Fungi DNA Isolation Kit (EURx Ltd., Gdańsk, Poland) was used, and the manufacturer’s recommended protocol was followed. The ITS1 (5′-TCCGTAGGTGAACCTGCGG-3′) and ITS4 (5′-TCCTCCGCTTATTGATATGC-3′) primer pair was selected for the amplification of the ITS region [32,33]. The PCR was carried out using Taq DNA polymerase; the reaction mixture consisted of 10× PCR buffer, MgCl_2_ (25 mM), dNTP mix (20 mM), primers, Taq DNA polymerase (2 U) and DNA template. The PCR cycle consisted of the following steps: an initial denaturation at 94 °C for 5 min; followed by denaturation at 94 °C for 30 s, annealing at the optimum temperature for 30 s, extension at 72 °C for 1 min, and finally a final extension at 72 °C for 10 min. The reaction was terminated indefinitely at 4 °C. The resulting PCR products were purified using the MAGBIO HighPrep PCR Clean-up System (MagBio Genomics, Gaithersburg, MD, USA); sequencing was performed bidirectionally using an ABI 3730XL (Applied Biosystems, Foster City, CA, USA) sequencer and the BigDye Terminator v3.1 Cycle Sequencing Kit. The resulting sequences were edited using BioEdit Sequence Alignment Editor version 7.2.5 [34] and assembled using the CAP contig assembly method. For species identification, the obtained sequences were compared with reference sequences in the GenBank database [33]. Type-derived ITS sequences were searched for both target species in GenBank and the UNITE database. As no type-derived ITS sequences were available for *Hypomyces aurantius* or *Lyophyllum littoralis*, representative reference sequences retrieved from GenBank were selected for phylogenetic analyses.

### 2.4. Extracellular Enzyme Production and Screening

An extracellular enzyme screen was carried out to evaluate the mycoparasitic activity of *H. aurantius* on its host fungus and its environmental survival strategies. To determine protease activity, media containing two different substrates (casein and gelatine) were used: Modified Casein agar (0.5% yeast extract, 0.03% calcium hydroxide, 0.02% calcium chloride, 1.5% agar, 1% casein, pH 5.8) [35] and Glucose Yeast Extract Peptone Agar with gelatine (1% peptone, 0.5% glucose, 2% glucose, 1.5% agar, 0.4% gelatine) [36]. To determine lipase activity, a medium supplemented with 1% tributyrin (0.5% peptone, 0.3% yeast extract, 2% agar, 1% tributyrin and 0.05% Tween 80) was used [37]. In the investigation of laccase activity, PDA media were used, both containing 0.15 mM CuSO_4_, with the addition of 2 mM ABTS and 4 mM guaiacol, respectively [38]. For the determination of amylase activity, a culture medium containing 0.1% glucose, 0.01% yeast extract, 0.05% peptone, 1.6% agar, and 0.2% soluble starch was prepared [36].

Mycelial discs, obtained from fresh *H. aurantius* cultures using a sterile mushroom corer (5 mm in diameter) were inoculated into the centre of the prepared culture media. All experiments were carried out in triplicate, and the Petri dishes were incubated at 25 ± 2 °C and observed daily. Protease, lipase and amylase activities were assessed as positive based on the presence of the formation of a halo around the colony. Laccase activity was determined by the characteristic green colour formation around the colonies resulting from the oxidation of ABTS and by the reddish-brown colour formation resulting from the oxidation of guaiacol. Following incubation, the casein agar plates were treated with a 1% HCl solution, whilst the gelatin-containing plates were treated with a saturated ammonium sulphate solution to cause opacification and render the hydrolysis zones around the colonies visible. In contrast, starch-containing media were flooded with an iodine solution, which stained the agar blue-black. Clear zones surrounding the colonies, where starch hydrolysis had occurred, were considered indicative of amylolytic activity [35,36,37,38].

The capacity for producing extracellular enzymes is expressed as the enzyme index (EI), which is calculated by dividing the total diameter of the reaction zone (i.e., the hydrolysis or colour change zone) by the diameter of the fungal colony. The formula is as follows [39]:

EI = Colony diameter/Diameter of reaction region including colony.

The diameters of both the colony and the hydrolysis zone were measured in millimetres using a digital calliper. Measurements were obtained from three independent replicates, and the results are presented as the mean ± standard deviation (SD).

## 3. Results

### 3.1. Morphological Results

Based on the taxonomic keys cited in the scientific references, the phylogenetic and morphological identification of *H. aurantius* and its host *L. littoralis*, which were collected and isolated from their natural habitat, along with brief descriptions of the specimens, macro- and micromorphological photographs, micromorphological drawings, and the collection site and date, ITS rDNA (nuclear internal transcribed spacer) gene sequences and phylogenetic analyses are presented below.

Ascomycota Caval.-Sm.

Sordariomycetes O.E. Erikss. & Winka

Hypocreaceae De Not.

*Hypomyces* Fr.

**Figure 2:**
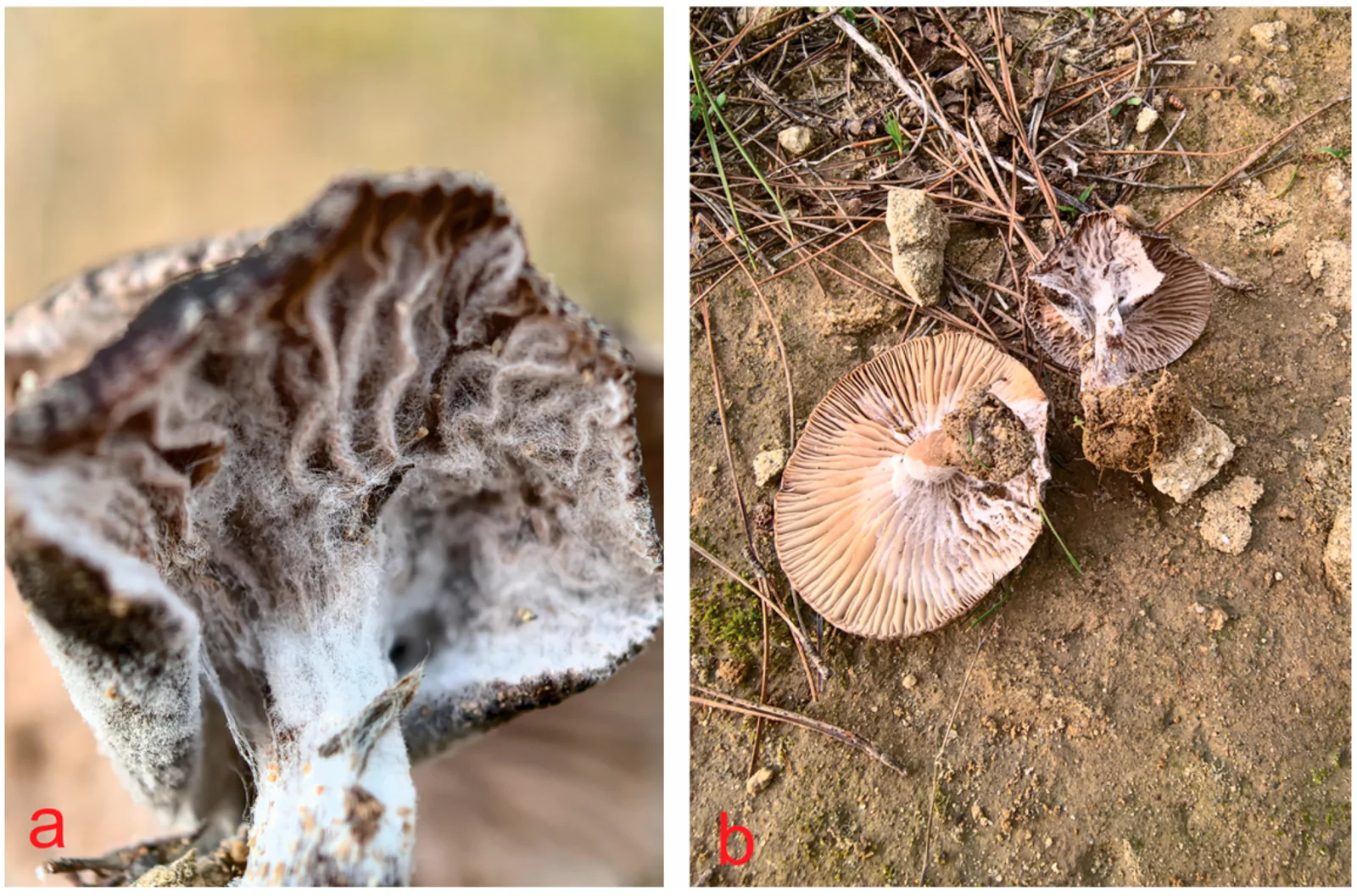
*H. aurantius* (**a**,**b**). On fresh basidiomata of *L. littoralis*.

*Hypomyces aurantius* (Pers.) Fuckel, *Jb. nassau. Ver. Naturk.* 23–24: 183 (1870) [1869–70] (Figure 2)

GenBank No: PV715959

**Figure 3:**
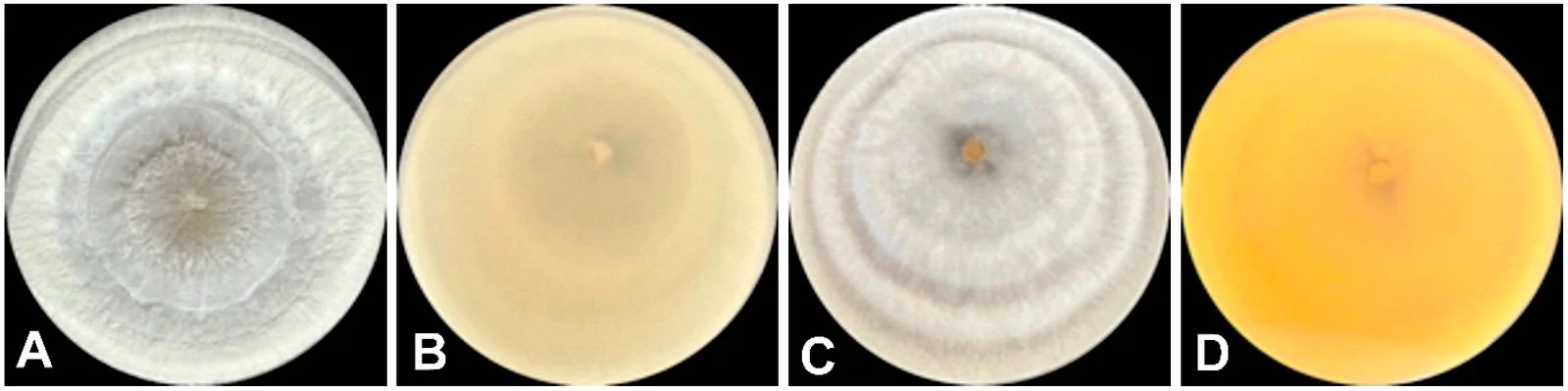
Colony morphology of *H. aurantius* showing concentric mycelial zones. (**A**,**B**) Top and bottom views of colonies on PDA medium after 5 days at 25 °C; (**C**,**D**) Top and bottom views of colonies on MEA medium after 5 days at 25 °C.

Colonial morph: Colony on PDA reaching 70–80 mm in diameter after 5 days at 25 °C, diffuse, cottony to floccose, with a well-developed aerial mycelium. The central region is compact and slightly raised (umbonate); abundant conidia imparting a powdery texture to the colony surface. Initially white to cream, becoming pale yellowish-brown with age. Reverse light beige to ochre, with distinct zonation (Figure 3).

**Figure 4:**
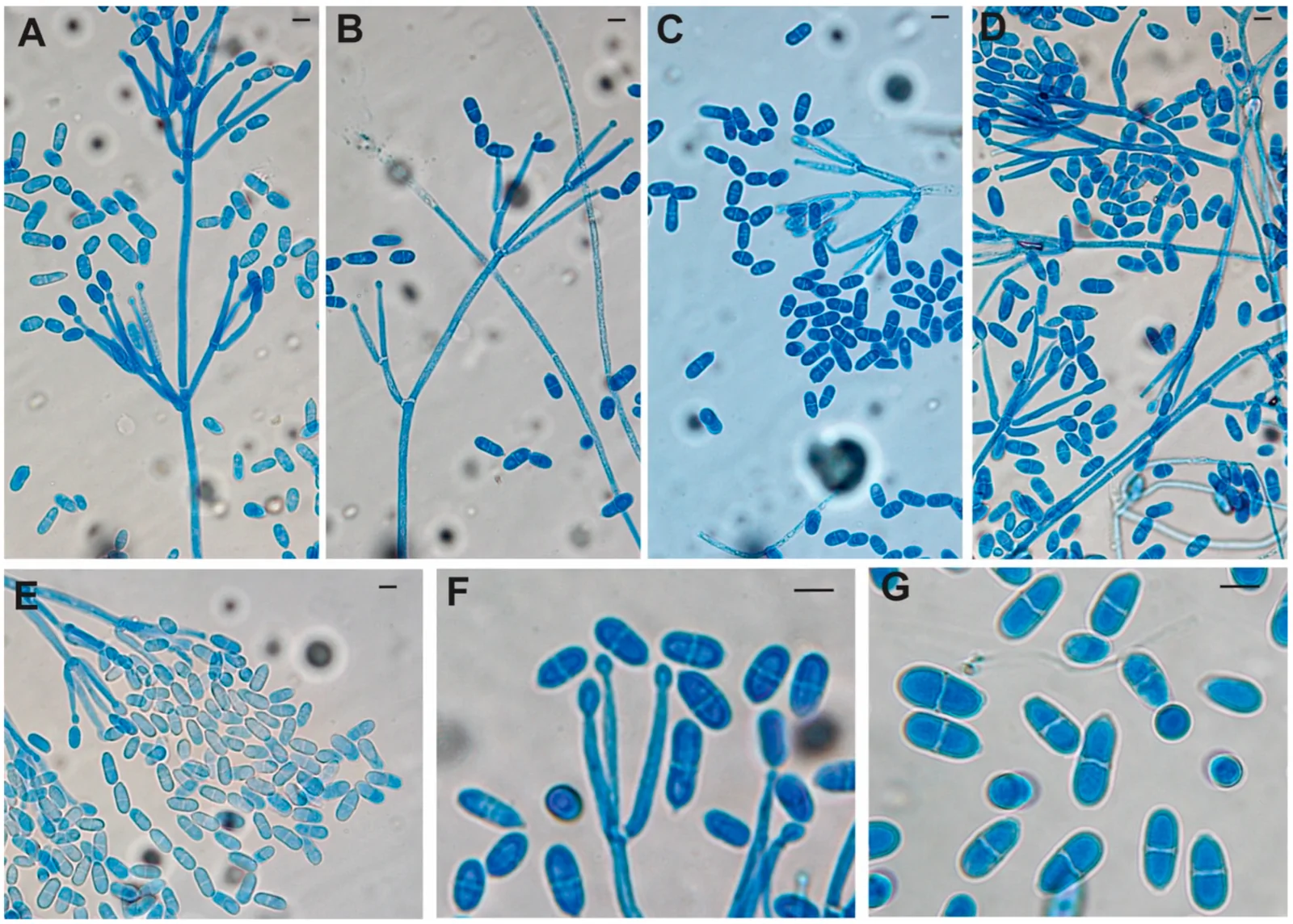
Micromorphology of *H. aurantius*. (**A**–**F**) Conidiophores with conidiogenous cells formed in verticils and conidia; (**G**) One-septate conidia. Scale bars: 10 μm.

*L. littoralis* was found growing on the fresh fruiting body. Asexual morph: White mycelium on the host fruiting body. Hyphomycetous growth was observed on PDA medium; the mycelium consisted of hyaline, septate and distinctly branched hyphae with straight walls. Conidiophores up to 90 μm in length, hyaline, with a structure that is either unbranched or branched vertically once or more, the branches tapering slightly towards the tip and terminating in conidiogenous cells (20–45 μm in length). Conidia 12.5–21.5 × 5.5–10.0 μm, initially spherical to hemispherical, becoming ellipsoidal to obovoid at maturity; smooth-walled, aseptate or rarely 1-septate, with a truncate basal hilum. Conidia formed singly and arranged in dry, basipetal chains joined end-to-end. Chlamydospores not observed. Sexual morph not observed (Figure 4).

Specimens examined: Türkiye, Çanakkale, Terzioğlu Campus, Near the Faculty of Science, 40°06′35″ N, 26°25′08″ E, 96 m, on fresh basidiomata of *L. littoralis*, 3 January 2025, HK001.

Distribution and habitat: *Hypomyces aurantius* have been reported from different geographical regions and is associated with a wide range of macrofungal hosts. It grows on the basidiomata of various fungi, including decaying polypores, *Panellus* spp., cultivated mushrooms, and other macrofungi reported in the literature. During colonization, it forms a characteristic orange-coloured mould layer on the host surface, frequently accompanied by discoloration, deformation, and progressive deterioration of host tissues [1,17,18].

Basidiomycota Whittaker ex R.T. Moore

Agaricomycetes Doweld

Agaricales Underw

Lyophyllaceae Jülich

*Lyophyllum* P. Karst.

**Figure 5:**
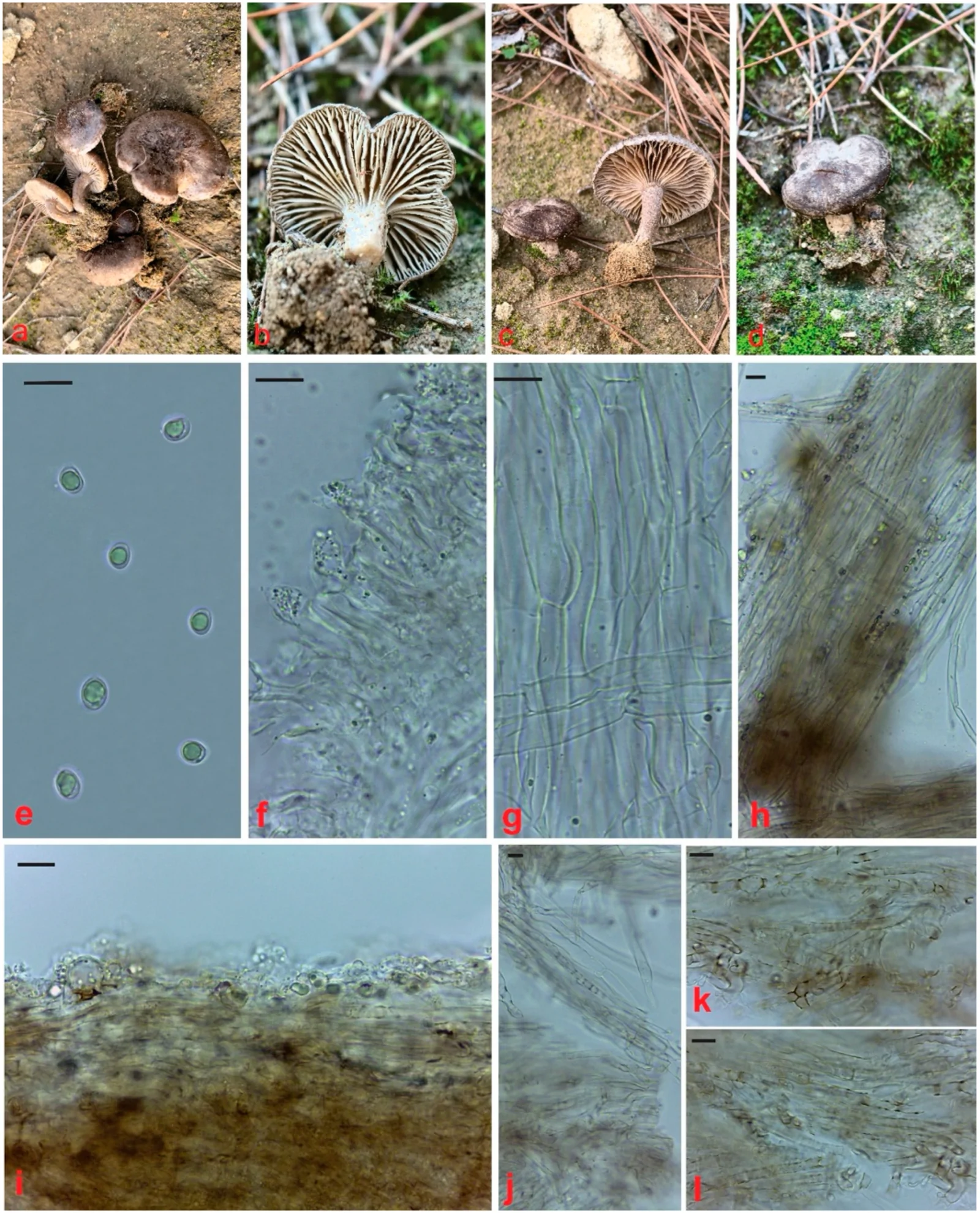
*Lyophyllum littoralis* (**a**–**d**). Fresh basidiomata in natural habitat, (**e**) spores, (**f**) basidia, (**g**) hyphae of lamellae, (**h**) stipipellis, (**i**–**l**) pileipellis. Scale bar: 10 µm.

**Figure 6:**
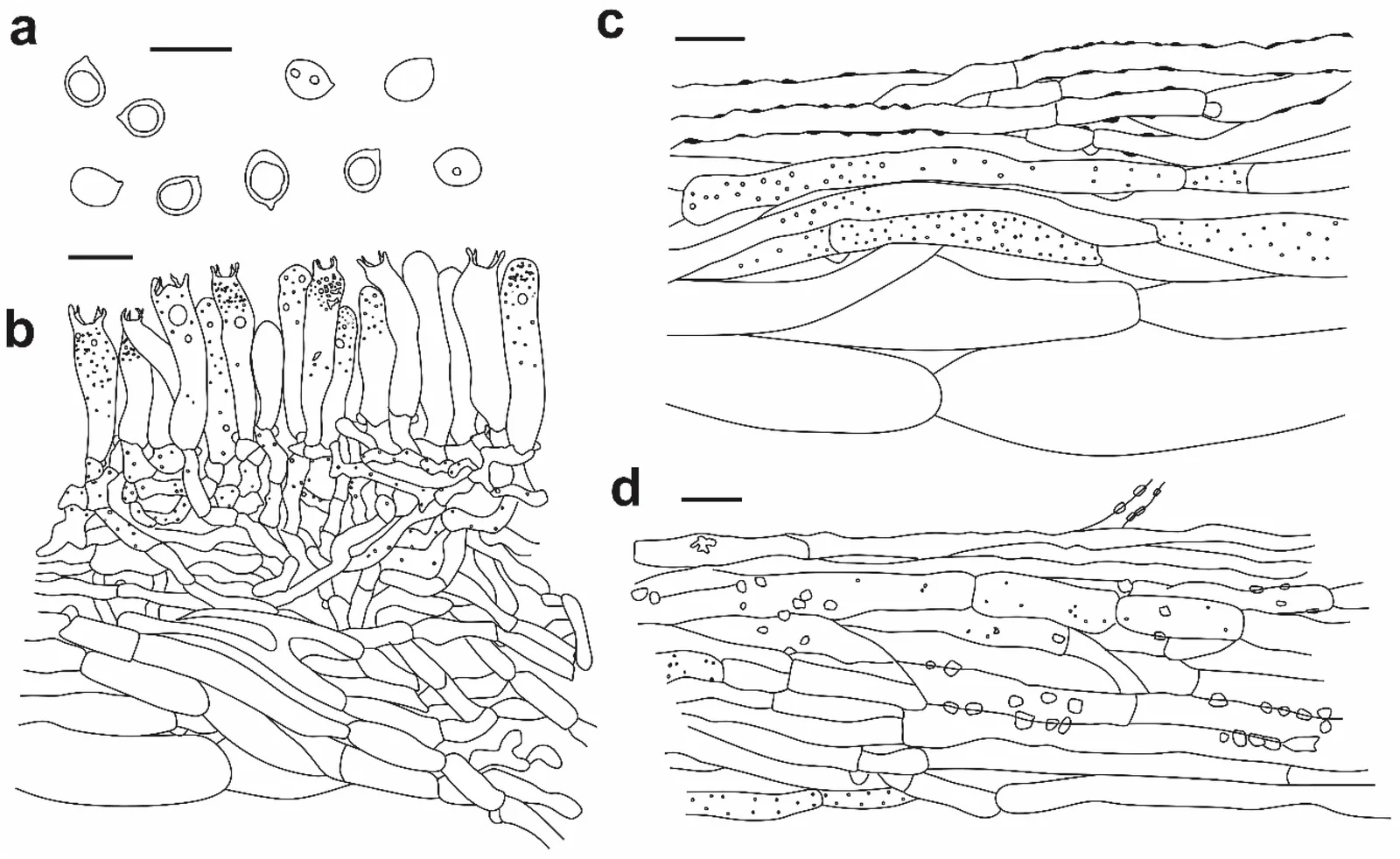
*L. littoralis* (**a**) basidiospores, (**b**) hymenophoral trama, (**c**) pileipellis, (**d**) stipipellis. Scale bar: 10 µm.

**Figure 7:**
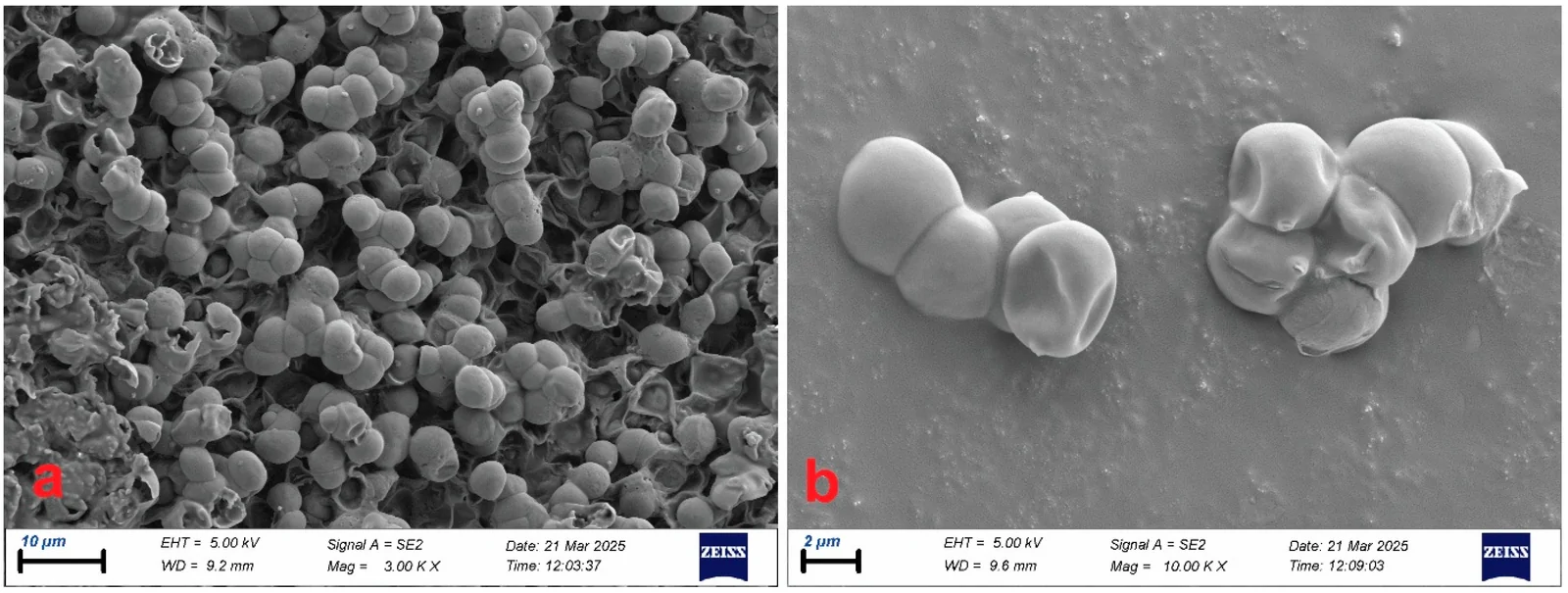
Scanning electron micrographs (SEM) of the basidiospores of Lyophyllum littoralis: (**a**) general view of basidiospores at 3.00 K× magnification; (**b**) detailed view of individual basidiospores at 10.00 K× magnification, showing their surface morphology. Scale bars: (**a**) 10 µm; (**b**) 2 µm.

*Lyophyllum littoralis* (Ballero & Contu) Contu, Boll. Gruppo Micol. G. Bresadola 41 (3): 193 (1998) (Figure 5, Figure 6 and Figure 7)

Basionym: *Calocybe littoralis* Ballero & Contu

GenBank No: PV715960

Pileus 30–100 mm, shiny to matte, spotted, brownish grey, sometimes slightly fibrillate from center to margins, pileus usually covered with a white powdery layer, margins darker, sometimes with brown transverse stripes on margins, pileus shape in young specimens usually rounded, curved when mature, margin curved downwards. Fleshy, thin, and rubbery, the stipe is not quickly broken; young specimens are generally odorless, while mature specimens emit a nutmeg odor. Stipe 15–35 × 4–10 mm, hollow, white or whitish fibrous near pileus, brown towards the base, grayish brown; in mature specimens, usually grey. Lamellae adnexed or adnate, white when young, pale yellowish gray with age, margin typically straight, sometimes slightly wavy. The short lamellae are of variable length and irregularly arranged between the main ones. Basidia (21.4–)24–32(–35) × 4.5–6.2(–7) µm, (n = 25), cylindrical to clavate, hyaline, with large or small drops, sometimes siderophilous granulation near the apex, with clamp connections. Marginal cells similar to basidia. Spores 4.8–6.2(–7.5) × 4.5–6 µm, (n = 30), av. 5.8 µm, globose to subglobose, smooth, hyaline, usually with a significant drop in the center, sometimes without a drop or with several tiny drops. Hyphae of lamellae up to 14 µm, hyaline, cylindrical, with basal clamp. Pileipellis, the uppermost layer, brown to yellowish brown incrusted, 3.5–6.5 µm wide, with clamp, middle layer siderophilous granulation, cylindrical, brown to yellowish brown, up to 15 µm, with clamp, lowest layer cylindrical parallel hyphae, smooth, hyaline, without granules, up to 18 µm wide, with clamp. Stipitipellis a cutis of elongate and crystalline hyphae, hyaline to yellowish brown, 2–6 µm wide. Clamp connection present at some septa.

Specimens examined: Türkiye, Çanakkale, Terzioğlu Campus, near the Faculty of Science, 40°06′35″ N, 26°25′08″ E, 96 m, under *Pinus brutia* Ten trees, 3 January 2025, Acar 1890.

Distribution and habitat: *L. littoralis* fruits in Mediterranean forests, especially under pine trees, between November and January. Although it usually develops in clusters attached to a common base, it can sometimes be seen as single individuals [22,24].

### 3.2. Phylogenetic Results

**Figure 8:**
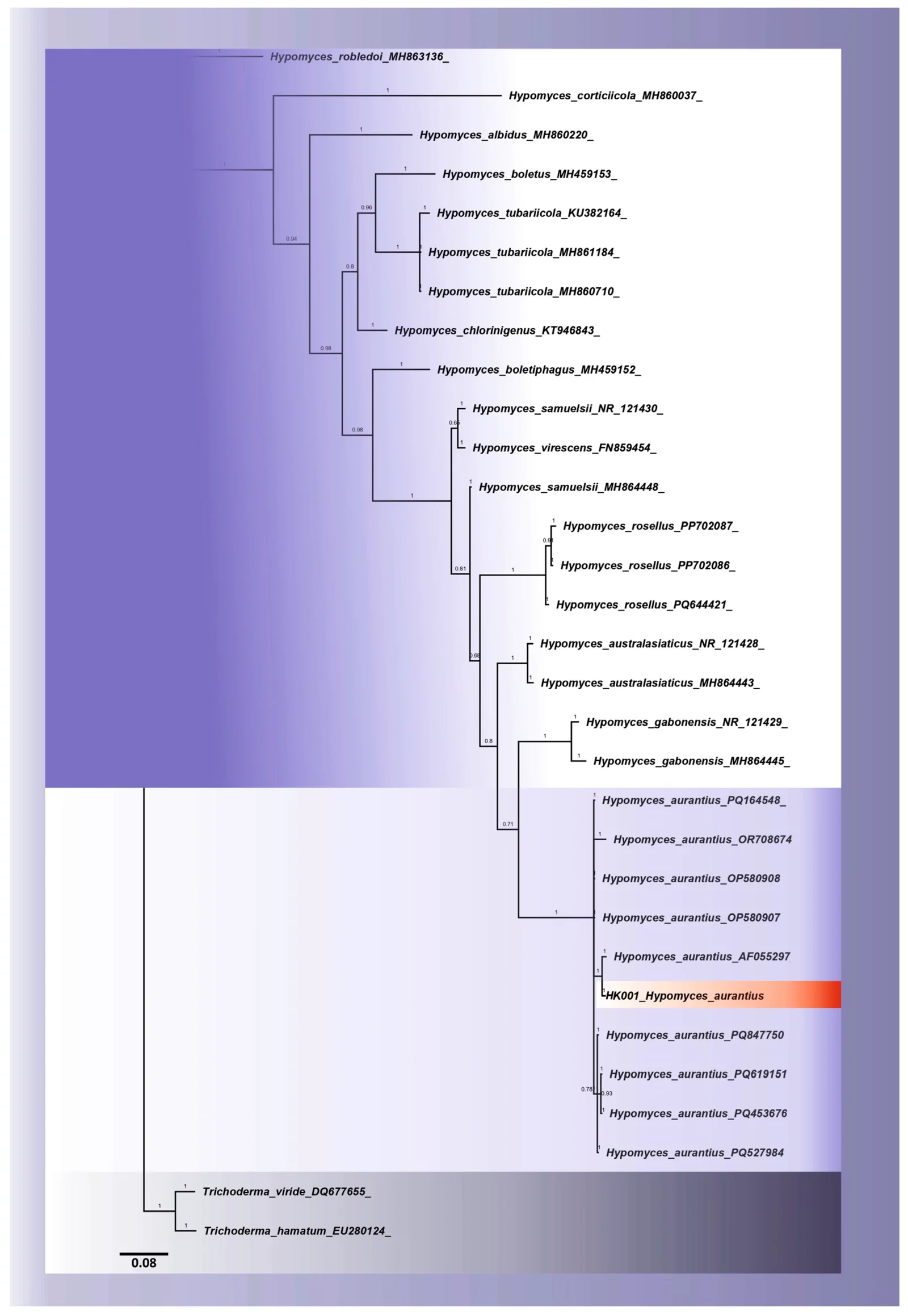
Bayesian inference (BI) phylogenetic tree of *Hypomyces* based on nrITS sequences. Bayesian posterior probability values (BPP ≥ 0.50) are shown at the nodes. The newly generated sequence HK001 (*H. aurantius*), highlighted in red, clusters within the *H. aurantius* clade. Background colors distinguish the major groups represented in the tree: the *H. aurantius* clade is shown in **light purple**, the remaining *Hypomyces* taxa in **purple**, and the outgroup taxa in **gray**. *Trichoderma viride* and *T. hamatum* were used as outgroups. The scale bar represents substitutions per site.

**Figure 9:**
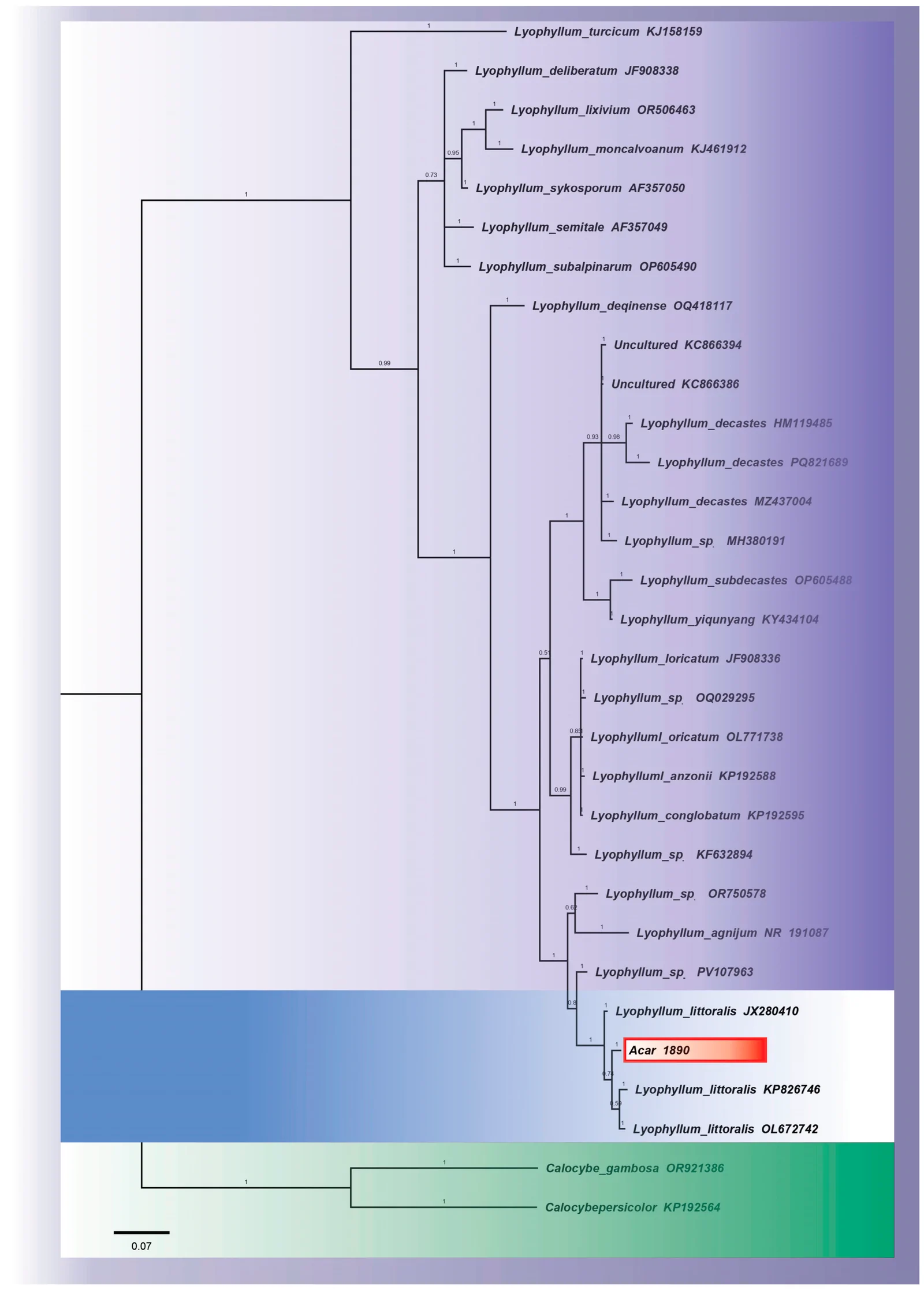
Bayesian inference (BI) phylogenetic tree of *Lyophyllum* based on nrITS sequences. Bayesian posterior probability values (BPP ≥ 0.50) are indicated at the nodes. The newly generated sequence Acar 1890 (*L. littoralis*), highlighted in red, clusters within the *L. littoralis* clade. Background colors distinguish the major groups represented in the tree: the *L. littoralis* clade is shown in **blue**, the remaining *Lyophyllum* taxa in **purple**, and the outgroup taxa in **green**. *Calocybe gambosa* and *C. persicolor* were used as outgroups. The scale bar represents substitutions per site.

The nrITS sequences obtained in this study were used to identify the two taxa under investigation at the molecular level. The newly generated sequences (PV715959 for *H. aurantius* and PV715960 for *L. littoralis*) were aligned with reference sequences from the GenBank database and included in Bayesian phylogenetic analyses. The *H. aurantius* (PV715959) sequence was positioned within the same clade as the reference *H. aurantius* sequences in the resulting phylogenetic tree, within a group supported by high posterior probability values. No topological deviation was observed within this clade, and the results clearly demonstrate that the species has been correctly identified at the species level. Similarly, the *L. littoralis* (PV715960) sequence was also placed within a well-supported clade alongside the reference *L. littoralis* sequences. In particular, the fact that the Acar 1890 specimen shows a direct sister-group relationship with the *L. littoralis* sequences reported in the literature strongly supports the species identification. Furthermore, the fact that this clade is distinct from other taxa within the *L. decastes* complex demonstrates that the species is molecularly distinguishable. The phylogenetic trees generated from both analyses consistently recovered the examined specimens within their respective species, with stable topological placement (Figure 8 and Figure 9). When evaluated in conjunction with morphological data, these findings demonstrate that the identification of both taxa has been carried out with a high degree of reliability.

### 3.3. Results for Extracellular Enzyme Production and Screening

**Figure 10:**
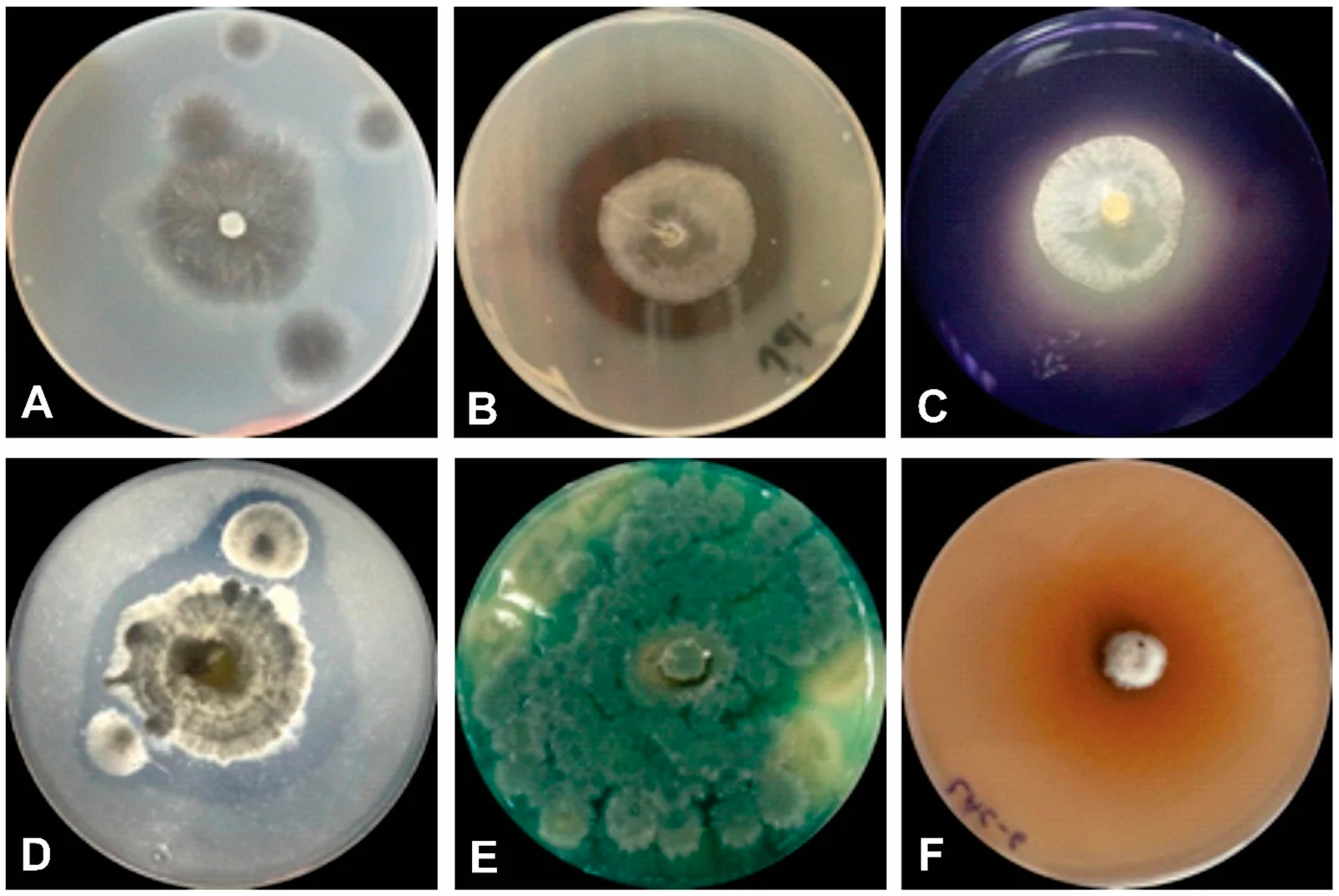
Representative Petri plate assays demonstrating extracellular enzymatic activities of *H. aurantius*. (**A**) caseinase, (**B**) gelatinase, (**C**) amylase, (**D**) lipase, (**E**) laccase activity on ABTS medium, and (**F**) laccase activity on guaiacol medium.

*H. aurantius* showed positive reactions for all tested extracellular enzymes (protease, lipase, amylase, and laccase) (Figure 10).

The extracellular enzymatic profile of *H. aurantius* was evaluated using qualitative plaque assays, and enzyme production was expressed as the enzyme index (EI) (see Table 1 for details). The isolate exhibited positive activity for all tested enzymes; however, the level of enzyme production varied according to the substrate. The results of the qualitative analysis demonstrated that the isolate was capable of producing both hydrolytic enzymes, including gelatinase, amylase and lipase, and oxidative enzymes, such as laccase.

## 4. Discussion

This study presents the first morphological and molecular characterization of *Hypomyces aurantius* and its host, *Lyophyllum littoralis*, two species newly recorded for the Turkish mycobiota. *Hypomyces aurantius* is a widely distributed fungicolous species that has been reported from numerous geographical regions worldwide. Despite its broad distribution, its host associations remain incompletely documented, and recent studies have demonstrated that the species is capable of colonizing a wider range of basidiomycete hosts than previously recognized. In the present study, *H. aurantius* was found on *L. littoralis* for the first time. This newly documented host association expands the known host range of the species and contributes to a better understanding of its ecological interactions and host adaptation.

The genus *Lyophyllum* Karsten belongs to the family Lyophyllaceae and comprises saprophytic agaric fungi found worldwide, characterised by white cyanophilic spores and siderophilic granules in their basidia [38]. The section Difformia (Fr.) Kühner, to which *L. littoralis* (Ballero & Contu) Contu belongs, is characterised by clustered fruiting bodies, does not turn brown, and is defined by its distinctive growth habits. It is currently known that *Lyophyllum* sect. Difformia comprises 15 caespitose and/or non-browning *Lyophyllum* species worldwide [40,41,42]. Some species of this genus, which plays an important role in natural ecosystems and possesses significant economic value, have been cultivated in Asia, the Americas, and Europe [43]. *Lyophyllum littoralis* is a Mediterranean species that typically grows in caespitose clusters in coniferous forests, particularly beneath *Pinus* spp. Morphologically, it closely resembles *L. decastes* but differs in its ecological preferences and geographical distribution, with *L. littoralis* occurring predominantly in warmer Mediterranean habitats, whereas *L. decastes* is associated with cooler temperate regions. The morphological characteristics and ITS-based phylogenetic placement of the Turkish specimen are fully consistent with these diagnostic features, supporting its identification as *L. littoralis*.

The genus *Hypomyces* was first described by Fries (1825) [44] as a subgenus of *Hypocrea* Fr., and was subsequently reclassified as an independent genus by Tulasne & Tulasne (1860) [19]. A comprehensive taxonomic study of this group was conducted by Arnold (1971) [45], who highlighted the distinguishing characteristics of *Hypomyces* and related genera. The life cycle of *Hypomyces* comprises anamorphic and teleomorphic reproductive stages. Many *Hypomyces* species in the asexual stage were previously assigned different scientific names and placed in different genera (*Cladobotryum*, *Mycogone*, *Sepedonium*, *Verticillium*, and others) [12,46]. Some *Hypomyces* species are rarely observed at certain stages of their life cycle. For example, whilst the sexual reproductive stage of some species that attack boletes is very rarely encountered, the asexual stage is rarely observed in other species. Furthermore, the anamorphic reproductive stage of many *Hypomyces* species remains unknown. This complicates the identification of species, as there are distinct morphological differences between the asexual and sexual stages. Consequently, the identification of *Hypomyces* species cannot always be based on consistent morphological characteristics. Nevertheless, they can be identified using molecular techniques and by the brightly coloured perithecia of species within the genus, which cause lesions and growth abnormalities on the host fungus [47,48].

Although the phylogenetic analyses of both species were based solely on the nrITS region, the obtained sequences showed high similarity to reference sequences and were consistently recovered within well-supported species-level clades in the Bayesian phylogenetic analyses. However, it has been reported that ITS data alone may not always be sufficient for a more reliable assessment of species boundaries within *Hypomyces* and the related *Cladobotryum* complex; therefore, multi-gene phylogenetic approaches incorporating additional loci such as LSU, tef1 and rpb2 may enhance taxonomic resolution [1,49].

In this study, mycoparasitic structures could not be directly visualised at the electron microscope level. One of the main reasons for this is the short duration of the active phase of the parasitism in the field and the difficulty in preserving suitable specimens in a manner appropriate for SEM analysis. However, SEM images of *L. littoralis* spores provided a clearer visualization of the species’ diagnostic characteristics.

*H. aurantius* is known in the literature as a species that exhibits mycoparasitic and pathogenic effects on various macrofungi. In particular, it has been reported to cause cobweb disease in morel species and to have a broad host range [50]. Cobweb disease is a serious fungal disease caused by *Hypomyces*/*Cladobotryum* species and characterised by a white, hairy mycelial network that spreads rapidly across the surface of the growing medium. The disease begins with the invasion of primordia, leading to discolouration and progressive rot in the fruit bodies [17,51]. It has been reported that *H. aurantius* parasitises *Panellus* species in China. In particular, it is known that such mycoparasites also cause infection in economically important species, such as the previously cultured *Flammulina velutipes* [18,52]. *H. aurantius* has also been reported on *Lenzites warnieri* in the Balkan Mountains [53,54]. It has been reported that *H. aurantius* on *Xylobolus subpileatus* constitutes the sole record on *Xylobolus*, a genus thought to be closely related to *Stereum*, as cited by [55,56].

The successful establishment of mycoparasitic fungi that colonise their fungal hosts is a complex biological process dependent on the coordinated secretion of extracellular hydrolytic and oxidative enzymes capable of breaking down the structural components of the host cell wall [57,58]. In this study, the protease, lipase, amylase and laccase activities identified in *H. aurantius* indicate that the species possesses an extracellular enzyme repertoire capable of supporting its fungicolous lifestyle. Similarly, studies conducted on various fungicolous fungi have shown that these organisms can commonly produce proteases, amylases, cellulases and various oxidoreductases, and that these enzymes may play a role in both nutrient acquisition and host colonisation [39]. The protease activities identified in our study suggest a biochemical capacity for facilitating hyphal penetration and nutrient acquisition through the hydrolysis of proteins in the host fungal cell wall and intercellular matrix. Similarly, lipase activity may support the colonisation process by contributing to the breakdown of lipid components in the cell membrane [57]. Laccase activity, on the other hand, demonstrates that *H. aurantius* possesses the capacity to produce not only hydrolytic but also oxidative enzymes. Although laccase is not an enzyme that directly breaks down the fungal cell wall, it may indirectly support host colonisation by contributing to processes such as the oxidation of phenolic and aromatic compounds involved in the host’s chemical defence, the conversion of organic matter, and the reduction in oxidative stress [59,60]. Amylase activity may contribute to the utilisation of carbon sources by facilitating the hydrolysis of storage polysaccharides found in host tissues or in surrounding organic material. Furthermore, this amylolytic capacity may indicate a metabolic flexibility that could support the organism’s survival outside the host on carbon-rich substrates such as soil organic matter or decaying plant residues [61,62].

However, chitinases and β-1,3-glucanases, which hydrolyze chitin and β-1,3-glucan, the major polysaccharides of the host fungal cell wall, have long been recognized as key enzymes in mycoparasitic fungi. In particular, it has been reported that enzymes that degrade β-1,3-glucan show a strong correlation with mycoparasitic success in fungicolous fungi, whereas cellulase production is not a determining factor for successful mycoparasitism [63]. Recent genomic studies also demonstrate that fungicolous fungi possess a rich repertoire of CAZyme enzymes and that these enzymes play a fundamental role in nutrient acquisition through the degradation of the host cell wall [4]. On the other hand, it has been shown that a fungicolous lifestyle does not always imply mycoparasitism; in some species, commensal relationships with the host fungus can develop despite chitinase and β-1,3-glucanase activities being quite low [64]. Consequently, the fact that chitinase and β-1,3-glucanase activities were not assessed in our study limits the ability to draw direct conclusions regarding the enzymatic mechanisms by which *H. aurantius* degrades the host cell wall. Nevertheless, the identified enzyme activities suggest that the species may possess the biochemical capacity necessary for host colonisation and nutrient acquisition. In future, investigating chitinase and β-1,3-glucanase activities in conjunction with biochemical assays, transcriptome analyses and CAZyme-based genomic approaches will contribute to a much more detailed understanding of mycoparasitic strategy of *H. aurantius* and its interaction mechanisms with *L. littoralis* [65,66].

The intense mycelial growth and marked softening and decomposition of the host tissue observed in this study are consistent with the symptoms reported in the literature. However, *L. littoralis* is a new host for this species, previously unreported, and is documented here for the first time. However, as experimental validation of pathogenicity could not be carried out, the interaction in question has been assessed as a ‘possible mycoparasitic relationship’. Consequently, this study not only contributes to Türkiye’s fungal biodiversity but also represents a step towards filling gaps in the literature regarding the documentation of fungicolous/possible mycoparasitic fungi in nature. Future multi-locus phylogenetic studies incorporating additional loci (e.g., LSU, tef1-α, and rpb2) together with type-derived reference sequences, where available, as well as culture-based isolations and controlled mycoparasitism experiments, will provide improved phylogenetic resolution and enable a more detailed understanding of the biology, species delimitation, and ecological roles of these species.

## Figures and Tables

**Table 1:** Qualitative extracellular enzyme activities of *H. aurantius* expressed as enzyme index (EI, mean ± SD, n = 3).

Enzyme	Substrate	EI (Mean ± SD)
Protease	gelatine	1.521 ± 0.04
Protease	casein	1.021 ± 0.01
Amylase	soluble starch	1.489 ± 0.03
Lipase	trybutirin	1.382 ± 0.05
Laccase	guaiacol	3.063 ± 0.06
Laccase	ABTS	1.401 ± 0.05

## Data Availability

The data supporting the findings of this study are available within the article. The newly generated nrITS sequences have been deposited in GenBank under accession numbers PV715959–PV715960. Additional data are available from the corresponding author upon reasonable request.
